# Do portfolios have a future?

**DOI:** 10.1007/s10459-016-9679-4

**Published:** 2016-03-30

**Authors:** Erik Driessen

**Affiliations:** Department of Educational Development & Research, Faculty of Health Medicine and Life Sciences, Maastricht University, Maastricht, The Netherlands

**Keywords:** Assessment, Portfolio, Competency-based education, Reflection, Medical education

## Abstract

While portfolios have seen an unprecedented surge in popularity, they have also become the subject of controversy: learners often perceive little gain from writing reflections as part of their portfolios; scholars question the ethics of such obligatory reflection; and students, residents, teachers and scholars alike condemn the bureaucracy surrounding portfolio implementation in competency-based education. It could be argued that mass adoption without careful attention to purpose and format may well jeopardize portfolios’ viability in health sciences education. This paper explores this proposition by addressing the following three main questions: (1) Why do portfolios meet with such resistance from students and teachers, while educators love them?; (2) Is it ethical to require students to reflect and then grade their reflections?; (3) Does competency-based education empower or hamper the learner during workplace-based learning? Twenty-five years of portfolio reveal a clear story: without mentoring, portfolios have no future and are nothing short of bureaucratic hurdles in our competency-based education programs. Moreover, *comprehensive* *portfolios,* which are integrated into the curriculum and much more diverse in content than reflective portfolios, can serve as meaningful patient charts, providing doctor and patient with useful information to discuss well-being and treatment. In this sense, portfolios are also learner charts that *comprehensively* document progress in a learning trajectory which is lubricated by meaningful dialogue between learner and mentor in a trusting relationship to foster learning. If we are able to make such comprehensive and meaningful use of portfolios, then, yes, portfolios do have a bright future in medical education.

## Do portfolios have a future?

It was at the turn of the millennium that I changed from law school and knowledge testing to med school and use of portfolios. The words that my roommate spoke to me on my last day in law school were: “Erik, I hope you are not taking those portfolios too seriously.” Now, 16 years on, I have not only completed a PhD on portfolios, but also introduced portfolios into several undergraduate and postgraduate programs, helped to develop an e-portfolio system and participated in countless numbers of workshops, lectures, master classes, symposia, webinars and training sessions on portfolios. In my faculty portfolios have had an erratic life: they swiftly blossomed during the infant years, then changed and partly disappeared during adolescence, to mature and become the central learning and assessment instrument in our longitudinal integrated clerkships and assessment programs.

Had I expected this when I closed the door to my office in law school? Definitely not! The future of portfolios proved to be more serious than my roommate and I had envisioned back in 2000. And today in 2015, portfolios’ future seems brighter than ever, with students, residents and doctors compiling evidence of their learning and performance in a portfolio as part of their daily ritual (Chertoff et al. 2015). This begs the question: will this global rush on portfolios perpetuate their presumed success in the future? In this paper it is argued that portfolios’ popularity, if left unbridled, might just as well contribute to their demise. The key leitmotiv throughout this paper is the question of whether portfolios have a future in health care education. The portfolio future question enables me to approach some bigger topics: competency-based education, reflection and assessment. In my reflections, I will draw on lessons learned from the literature and intertwine these with personal anecdotes from my portfolio trajectory. It is beyond the scope of this paper to discuss the practical aspects of a portfolio, such as how to construct and implement an effective portfolio or how to assess a portfolio. I have extensively discussed these aspects elsewhere (e.g., Driessen and van Tartwijk 2013; van Tartwijk and Driessen 2009).

Other than the main question may suggest, I do not pretend to be able to predict the *future*, especially since portfolios have appeared to be capricious and unpredictable. Therefore, my reflections will center on three critical issues that surround portfolio:

- Why do portfolios meet with such resistance from students and teachers, while educators love them?
- Is it ethical to require students to reflect and then grade their reflections?; and
- Does competency-based education empower or hamper the learner during workplace-based learning?

In the next section I will discuss the different *portfolio* variations that enjoy currency.

## Different variations of a portfolio

What do you see when you envision a *portfolio*? This question will most probably elicit different answers from a student in Buenos Aires, a general practitioner in Leeds or a resident in Ottawa. Portfolios in fact differ so much in their form and use that it is almost impossible to make general statements about them. Despite their many variations, however, we can distinguish two overarching types, specifically the *reflective* portfolio and the *comprehensive* portfolio (Roberts et al. 2014). It is for comprehensive portfolios that I do see potential, although I am less convinced of the viability of reflective portfolios. Before delving into the whys and wherefores of these statements, I will first elucidate both variations.

### Reflective portfolios

The *reflective portfolio* is largely aimed at the development of reflective skills. It requires students to write up a reflection on an aspect that is considered important to their learning or profession. Reflective portfolios are either a self-contained part of the curriculum or added as an assignment to an already existing course. In my first publication on portfolios, I presented an example of such a portfolio, which required first-year students in Maastricht to reflect on four roles based on the assessment feedback they received (Driessen et al. 2003). While Roberts et al. (2014) use the term *formative* portfolio to refer to this type of portfolio, in my view this is confusing: in most cases progression to the next study phase is contingent upon successful conclusion of the portfolio. Another reason why I prefer not to distinguish between formative and summative assessment is because this distinction is less clear than one would think at first sight. For a critical perspective on this topic I refer to Man Sze Lau’s “Formative is good, summative is bad” paper (2015). Hence, for the purpose of this paper the term *reflective portfolios* refers to portfolios with a strong focus on reflection that are either self-contained or an addition to an existing course.

### Comprehensive portfolios

*Comprehensive* *portfolios* are integrated into the curriculum, i.e., they form part of an assessment program (Eva et al. 2015; van der Vleuten et al. 2012). This can be an undergraduate preclinical assessment program (Daneffer and Henson 2007) or a national postgraduate competency-based assessment program (Moonen-van Loon et al. 2013), for example. In these programs the portfolio is used in combination with other instruments to realize the program’s purpose. The portfolio brings together all the information that the other instruments have generated. The goal of comprehensive portfolios is twofold: to support the student’s learning process and to assess the student’s progress. Comprehensive portfolios can contain reflections, but their content is much more diverse than that of reflective portfolios.

Though other forms of portfolio exist, such as dossier types of portfolios or project portfolios, these are less common in health care education. The next sections will address three issues that are critical to the viability of portfolios in health care education.

## Public relations

*A portfolio would better suit my sister*

(de Hoog 2004, p. 7)

The most important issue we must address to prevent portfolio’s demise is its bad reputation. The last two decades have been characterized by happy portfolio developers and grumpy portfolio users. The enthusiasm and perseverance of educators introducing portfolios have met with stiff opposition from students and teachers working with these portfolios. Scientists, like me, have sought to investigate this resistance for many years (Driessen et al. 2007). We found that portfolios do not work by themselves. An important characteristic of portfolios is their vulnerability: they only work if several conditions have been fulfilled, e.g., mentoring, open structure, supporting learning environment and a direct learning gain for their users (Driessen et al. 2005). In the case of reflective portfolios this latter condition appears almost impossible to satisfy. Many students do not value reflection as a learning strategy, especially not when they are forced to use an artificial and fixed format and they have little opportunity to direct their learning as a result of these reflections. Most preclinical learning is structured and there is limited flexibility to adapt the environment to the student’s learning needs. All this causes students and teachers to be unsatisfied with their reflective portfolio, and, consequently, to become less motivated and engaged (Arntfield et al. 2015). As a result, they regard portfolios as just another assignment they have to do. Therefore, I do not believe that reflective portfolios are the way of the future, especially not when considering the problems involved in requiring students to reflect and assessing these reflections. The next section will look into this.

## Confessions

*Few things seem to irritate doctors and medical students so much as mandatory reflection.*

(Tomlinson 2015)

The surge of portfolios has gone hand in hand with the triumphal march of reflection into medical education. Portfolios are often synonymous with reflection, and, similar to portfolios, there are no signs of this triumphal march grinding to a halt. In Canada, physicians are expected to reflect regularly on their performance using various internal and external data sources (Royal College of Physicians and Surgeons of Canada 2015). In Belgium, moreover, besides reflecting on their performance, physicians are required to reflect on their professional attitude, the attitude and behavior of others, legal implications of patient care, and professional, ethical and legal codes (Michels et al. 2012).

While reflection is a valuable competence, it has also become a popular learning method in competency-based education. From the first introduction of competency-based learning students and residents fiercely resisted mandatory reflection as part of their training and assessment program. Scholars questioning current application of reflective practice have allied themselves with the reflection-tired students and residents. One of these dissenters, Brian Hodges, voices concern over the way we navigate between examination and reflection in medical education (2015). He worries about “the growing tension between high stakes external examinations driven by a discourse of ‘accountability’ and a more recent, but no less passionate, investment in internally motived notions of ‘self-direction’ and ‘reflection’” (Hodges 2015, p. 261). Mixing reflection with assessment leads, in Hodges’ view, to reflection as *confession*: the student reflects (confesses) and an external judge (confessor) “guides and shapes the accuracy and objectivity of the student’s reflection.” How harmful is it to force students to disclose personal feelings in their reflective portfolios and have these feelings assessed by a sometimes unknown assessor? (Ghaye 2007)

I too am worried about the unintended consequences of mandatory reflection. At the same time I am convinced that reflection in medical education has merits. In an earlier publication I endorsed Dewey’s definition of reflection as letting “your future behavior be guided by systematic and critical evaluation and analysis of actions and beliefs and the assumptions that underlie them” (Driessen et al. 2008, p. 827). This form of reflection is essential for learning from clinical experiences and therefore every learner in health care education should practice it. I also subscribe to Ng et al.’s assertion that reflection should more be seen as a critical social inquiry, requiring “more explicit attention to social and systemic forces, and the assumptions embedded in thought processes and power relations, with an aim toward transformation and action” (Ng et al. 2015, p. 465). Hence, it is my view that critical reflection could help to change medical education practice.

In sum, reflection places me in a dilemma: while I recognize its importance to learning and practice, I also seriously doubt mandatory pre-structured reflection. I don’t know the answer to this dilemma. A learning environment and a portfolio that values a reflective dialogue with a trusted person in an open and safe way, is probably the way to go. However, it is a long road for medical education to create such an environment. The learning environments and portfolios we currently offer to our learners are strongly focused on accountability and checking with all the unintended effects as a result.

## Kafka at the hospital

*When educators in graduate medical education first focused on competency-based education, I remember a rush to find assessment instruments. I struggled to have those same educators pause long enough to think about how they would teach the competencies they planned to assess.*

(O’Sullivan Commentary 2015, p. 277)

In competency-based learning portfolios have become instruments for the recording of large quantities of detailed outcomes, competencies, entrustable professional activities and milestones. At the end of the training period students have to prove that they have met all the requirements and their teachers have to check this. In time-scarce clinical practice the limited time for teaching is used for checking portfolio checklists instead of discussing feedback and learning. In this way portfolios degenerate into bureaucratic exercises instead of empowering our learners in the workplace. Rather than supporting the learning process, bureaucratic portfolios and other workplace-based assessment methods often stand in between the learner and the learning opportunities in the workplace (Teunissen and Eppich in press). I do not think this situation is likely to change in the near future. Educators are still working hard to develop outcomes, competences, entrustable professional activities and milestones, and the focus is mainly on assessment and less on how these outcomes can be learned. In the next section I will explain how I think comprehensive portfolios can contribute to the learning of outcomes and how we may be able to overcome the bureaucratic side effects of competency-based learning.

## Food for thought

While portfolios have seen an unprecedented surge in popularity, they have also become the subject of controversy. On all continents students, residents, physicians and other health care workers have adopted the practice of compiling a portfolio in their competency-based curricula. Yet, they often perceive little gain from their r*eflective* portfolios, while scholars question the ethics of obligatory reflection in *reflective* portfolios. Students, residents, teachers and scholars alike condemn the bureaucracy surrounding the implementation of *comprehensive* portfolios in competency-based education.

Is there a way out?

It is with good reason that the portfolio pioneers in medical education spoke of *portfolio learning* or *portfolio*-*based learning* instead of *portfolio assessment* (Snadden et al. 1996; Snadden and Thomas 1998) and defined portfolio as a “system [that] operates … through the interaction of a learner and supervisor using the material as a *catalyst* to guide [discussion and] further learning” (Snadden et al. 1996, p. 148). Hence, portfolios at best provide learners and their mentors with *food for thought*, which in the case of c*omprehensive* portfolios can be especially rich, with information and feedback on learning and past performance, the learner’s perspective on this combined with the learner’s intentions and plans for the coming period. These materials can feed the conversations between learners and mentors about performance, reassurance, and directing future learning and practice (Teunissen and Eppich in press). In the view of Eva et al. (2015) such a continuous model of assessment could help to overcome the unintended consequences of competency-based assessment, as it allows *inter alia* for competence to be closely monitored. Moreover, they prudently advise that *in an ideal world* a coach should discuss plans for improvement with the learner. For me, this addresses the root cause of the problem, as we are committed to accountability, define outcomes and milestones and develop instruments to measure them, but we pay no heed to learning; only in the *ideal world* do we ensure mentor time for every learner. This is unjust, for mentoring is likely the most powerful learning method available in our medical education toolbox (Driessen and Overeem 2013). Studies have reported beneficial effects of mentoring in both medicine and medical education, with mentoring having positive effects on career success, productivity, job satisfaction, career preparation, and workplace-based learning (Eby et al. 2008; Driessen and Overeem 2013).

Twenty-five years of portfolio reveal a clear story: without mentoring, portfolios have no future and are nothing short of bureaucratic hurdles in our competency-based education programs. Let us therefore commit ourselves to our learners. Let us establish a portfolio that supports learning by guiding the discussion between the learner and the mentor. Let us provide them with mentors who help them to use the portfolio data to make sense of their experiences and frame plans for improvement (Eva et al. 2015). Let us use the portfolio just as the patient chart. It provides doctor and patient with very useful information to discuss well-being and treatment. Let it also be a learner chart that *comprehensively* documents progress in a learning trajectory which is lubricated by meaningful dialogue between learner and mentor in a trusting relationship to foster learning. Recent calls for programmatic assessment cannot succeed without such a learning chart (van der Vleuten et al. 2012; Driessen et al. 2012). In programmatic assessment decision-making is removed from individual assessments and decisions are only based on longitudinally gathered assessment and learning information. The portfolio, then, becomes the key instrument that fully services learning and assessment. If we are able to make such comprehensive and meaningful use of portfolios, then, yes, I do envision a bright future for such learner charts in medical education.
